# Climate-Driven Habitat Redistribution and Conservation Gaps of the Medicinal Fern *Sceptridium ternatum* in China

**DOI:** 10.3390/biology15151268

**Published:** 2026-08-02

**Authors:** Jinchuan Guo, Yunyun Sun, Xinyu Zhang, Wanning Zhang, Lijuan Lian, Jiachen Sun, Zhiyuan Zhang, Junye Huang, Yating Wu, Qingshan Yang

**Affiliations:** 1 College of Pharmacy, Anhui University of Chinese Medicine, Hefei 230012, China; guojinchuan22@gmail.com (J.G.); syy045881@163.com (Y.S.); zhangxy0730420@163.com (X.Z.); 18994135785@163.com (W.Z.); 18356769112@163.com (L.L.); 18865052110@163.com (J.S.); 19334066472@163.com (Z.Z.); 15395587730@163.com (J.H.); 17855340310@163.com (Y.W.); 2 Anhui Province Key Laboratory of Research & Development of Chinese Medicine, Hefei 230012, China; 3 Institute of Conservation and Development of Traditional Chinese Medicine Resources, Anhui Academy of Chinese Medicine, Hefei 230012, China; 4 MOE-Anhui Joint Collaborative Innovation Center for Quality Improvement of Anhui Genuine Chinese Medicinal Materials, Hefei 230012, China

**Keywords:** ecological niche modeling, MaxEnt, CMIP6, spatial-block cross-validation, human footprint, protected-area coverage, germplasm survey

## Abstract

Climate change may redistribute understory medicinal plants, but national predictions are useful when model uncertainty and field constraints are explicit. We aimed to identify the current and future suitable habitat of *Sceptridium ternatum* and translate predictions into survey and conservation priorities. We compiled 187 occurrences, screened 104 candidate predictors to 9, calibrated MaxEnt with kuenm, and evaluated it using partial ROC, omission rate, AICc, random and spatial-block cross-validation, and the Boyce index. Future suitability was projected with two GCMs under SSP1-2.6 and SSP5-8.5. October precipitation had the highest permutation importance, and growing-season thermal accumulation was another major contribution. NDVI had high percent contribution but no unique permutation importance. The selected model achieved mean random-fold and spatial-block test AUCs of 0.9217 and 0.8575, respectively, and a Boyce index of 0.8211. Late-century SSP5-8.5 reduced total and highly suitable habitat by 45.06% and 89.96%, respectively. Protected areas covered only 14.77% of highly suitable habitat. The resulting framework separates regional climatic suitability from habitat compatibility and management feasibility, a distinction relevant to other data-limited understory medicinal plants. It distinguishes sites for population verification, germplasm collection, and long-term monitoring, and plot-scale decisions require measurements of canopy, moisture, substrate, population state, and mycorrhizal context.

## Simple Summary

Climate and land-use change may reduce habitat for *Sceptridium ternatum*, a winter-green medicinal fern of shaded forest and shrub understories. We mapped current and future suitability across China using 187 records and an optimized MaxEnt model. October precipitation was the strongest independent predictor, and broad vegetation greenness contributed to model fitting but did not measure local shade. Under a high-emission pathway, especially high-suitability habitat declined strongly by the end of the century, while existing protected areas covered only a small portion. We, therefore, separated protected monitoring sites, unprotected conservation gaps, and road-accessible survey areas. The maps are national screening tools rather than proof of local occupancy because canopy openness, soil moisture, population stage, and mycorrhizal conditions were not available nationally at the modeling resolution. Field teams should use the priority zones to verify populations, collect vouchers and germplasm, record microhabitat conditions, and establish repeated monitoring.

## 1. Introduction

Global climate change is altering plant range boundaries, phenology, and community composition [1], and many species have shifted toward higher latitudes or elevations [2]. For wild medicinal plants, climate-driven changes in temperature and moisture may interact with habitat conversion, harvesting, road construction, and insufficient protected-area coverage, thereby affecting population persistence, resource surveys, and subsequent use. Identifying present habitat suitability, future change trajectories, and conservation gaps is, therefore, an important basis for medicinal-resource surveys, germplasm collection, and in situ conservation in China.

Species distribution models (SDMs) estimate environmental suitability in unsurveyed areas from known occurrence records and environmental predictors [3]. Maximum entropy modeling (MaxEnt) requires presence and background data and is widely used for medicinal and rare plants, including species with relatively few records [4]. However, default settings may generate unnecessarily complex responses and increase uncertainty during model transfer [5]. The regularization multiplier (RM) and feature classes (FCs) directly affect model complexity and predictive performance [6]. Calibration tools, such as kuenm, permit candidate models to be compared using statistical significance, omission rate, and the corrected Akaike information criterion (AICc), thereby balancing fit and parsimony [7].

Recent ecological niche modeling studies increasingly combine parameter calibration, multi-source predictors, and explicit representation of projection uncertainty [8,9]. Random cross-validated area under the receiver operating characteristic curve (AUC) alone does not fully represent spatial transferability because it is affected by study extent, background sampling, and spatial autocorrelation [10]. The continuous Boyce index evaluates whether occurrence frequency increases with predicted suitability [11,12], while spatial-block cross-validation tests transfer to spatially independent regions [13,14]. Moreover, environmental suitability is not equivalent to habitat that a species can use. Land cover constrains the availability of compatible habitat [15], whereas human pressure and protected-area coverage influence conservation and survey priorities [16,17].

*Sceptridium ternatum* (Thunb.) Lyon is a small winter-green terrestrial fern treated in Flora of China under the synonym *Botrychium ternatum* and recorded beneath shrubs and in shaded habitats at 400–1000 m across many provinces of China and other East Asian regions [18]. These records describe its broad habitat but do not resolve canopy openness, forest stratum, soil moisture, substrate, or associated vegetation at individual sites. The species is used in traditional Chinese medicine for respiratory-related applications, and extracts have shown antitussive and antiasthmatic activity [19]. Unlike medicinal plants with established cultivation systems, *S. ternatum* currently lacks a stable large-scale cultivation or artificial supply basis. Wild populations and compatible habitats, therefore, remain the principal targets for resource assessment, germplasm collection, and in situ conservation.

Roots of *B. ternatum* have been reported to contain both orchid-type and arbuscular mycorrhizal associations [20]. More broadly, *Ophioglossaceae* can have subterranean gametophytes and early sporophytes [21], and genus-level evidence in *Sceptridium* shows shifts in fungal associations from fully mycoheterotrophic gametophytes to photosynthetic sporophytes [22]. These life-history features indicate that aboveground frond records may not fully describe population status and that local occupancy can depend on biotic and microsite conditions that regional climatic rasters cannot represent. However, the potential distribution, response to future climate change, and conservation gaps of *S. ternatum* have not been evaluated systematically across China.

The broader problem extends beyond this species. For data-limited understory medicinal plants, coarse regional suitability cannot be equated with locally usable habitat because canopy structure, vegetation stratum, soil moisture, biotic associations, harvesting pressure, and protection status can constrain occurrence after climatic screening. A transferable assessment should, therefore, evaluate model complexity and spatial transferability, then treat habitat compatibility and management feasibility as separate post hoc questions rather than interpreting a MaxEnt surface as a direct conservation decision [3,10,16,17].

We evaluated three working hypotheses: (H1) seasonal moisture and thermal accumulation would provide more independent information on regional suitability than broad vegetation greenness, (H2) high-emission climate change would reduce highly suitable habitat more strongly than total suitable habitat and redistribute its geographic centroid, and (H3) protected areas would cover only part of the highly suitable habitat, so land cover, human footprint, and road-distance overlays would distinguish conservation priorities from survey-accessibility priorities. Accordingly, we (1) calibrated a MaxEnt model using multi-source environmental predictors, (2) evaluated it using random and spatially structured validation, (3) projected suitability under two emissions pathways, two GCMs, and three future periods, and (4) translated current predictions into explicit conservation and field survey classes.

## 2. Materials and Methods

### 2.1. Occurrence Data and Spatial Thinning

Occurrence records were compiled from the Global Biodiversity Information Facility (GBIF; download DOI: 10.15468/dl.7fwvw5; download key: 0005384-260623161305970) [23], the Chinese Virtual Herbarium (CVH), PlantPlus, the National Plant Specimen Resource Center, the herbarium/specimen collection of the Chinese Materia Medica Resources Center at Anhui University of Chinese Medicine (ACM), the Fourth National Survey of Chinese Materia Medica Resources, and field investigations. Records with original coordinates were checked against their stated administrative locations. Records reported only to county, township, or locality level were georeferenced using the original specimen or survey description, administrative boundaries, and public gazetteers. Gazetteers were used only for coordinate conversion and location verification, not as occurrence sources. County- and township-level coordinates were flagged as lower precision and were not interpreted as exact collection locations.

**Figure 1:**
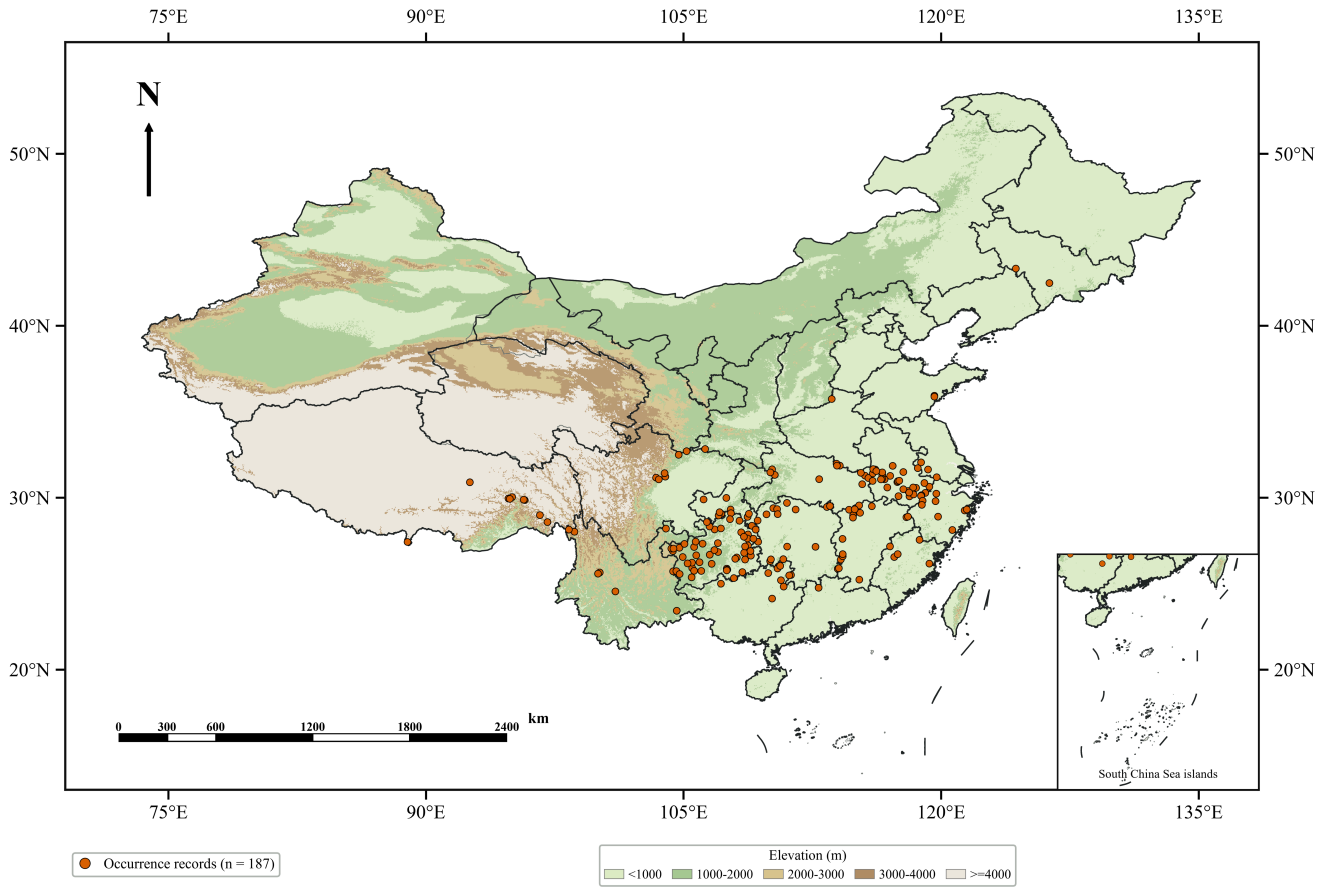
Occurrence records of *Sceptridium ternatum* and topographic background of the study area. Orange points indicate the 187 records retained after verification, coordinate checking, spatial thinning, and predictor-availability screening. Elevation is grouped into broad 1000 m classes. The inset identifies the South China Sea islands and is not an enlarged occurrence area.

The initial dataset contained 222 records. Duplicate records, clearly erroneous coordinates, and records with insufficient locality information were removed. Records were then spatially thinned at the 2.5 arc-min environmental-grid resolution (approximately 5 km), retaining one record per grid cell. Spatial filtering reduces sampling bias caused by clustered observations [24] and follows the rationale implemented in packages such as spThin version 0.2.0 [25]. Thinning retained 189 records, and 2 fell in cells lacking valid values in at least 1 final predictor and were excluded. The final modeling dataset comprised 187 records (Figure 1 and Table S1).

### 2.2. Environmental Predictors and Preprocessing

The 104 candidate predictors were assembled before screening to represent regional water and energy availability, seasonality, thermal accumulation, topography, substrate, vegetation background, and UV-B exposure. This intentionally broad candidate pool represented plausible regional environmental dimensions before transparent reduction—it was not treated as 104 direct physiological hypotheses or as evidence of causality. CHELSA v2.1 and CHELSA-BIOCLIM+ v2.1 provided bioclimatic, monthly climatic, growing-degree-day, and frost-related predictors [26,27]. Elevation was obtained from WorldClim v2.1, and slope and aspect were derived from the digital elevation model in ArcGIS 10.8. Soil attributes were obtained from the Harmonized World Soil Database v1.2 [28], ultraviolet-B predictors from glUV [29], and NDVI from the 2000–2022 China 30 m annual maximum NDVI dataset produced from Landsat 5/7/8/9 imagery [30]. The climatic and growing-degree-day variables represented seasonal moisture and thermal duration, topographic and soil variables represented broad terrain and substrate conditions, NDVI represented regional vegetation productivity, and UV-B represented regional radiation background. No temporally matched national layer of canopy openness, forest stratum, or understory shade was available at 2.5 arc-min. NDVI was, therefore, not treated as a direct measurement of shade. All predictors were projected to WGS 84 [31], resampled to 2.5 arc-min, clipped to the same extent, and aligned to a common grid.

Predictor reduction was performed in two explicit stages. First, a preliminary MaxEnt run containing all 104 variables was used as a pragmatic dimension-reduction step. Variables with percent contribution > 1.3% were retained, reducing the set from 104 to 11. This cutoff was an algorithmic screening rule rather than a physiological threshold. Second, pairwise Pearson correlations were calculated among the 11 variables. When |r| ≥ 0.8, the variable with the lower preliminary contribution was removed [32]. CHELSA BIO17 and BIO19 were excluded because they were strongly correlated with February precipitation (r = 0.935 and 0.986, respectively), yielding nine final predictors. Their pairwise |r| values were <0.8 and variance inflation factors ranged from 1.21 to 2.36 (Table S2). Elevation was included in the candidate pool but contributed 0.41% in the preliminary run and did not pass the first screen. A later diagnostic audit found substantial correlation between elevation and maximum-season UV-B (r = 0.873), but this redundancy does not imply that elevation is ecologically unimportant or that UV-B is a direct substitute for elevation. Table S2 now reports the preliminary contribution, screening decision, correlated partner where applicable, and final status for every candidate variable. The candidate groups and final retained variables are summarized in Table 1.

### 2.3. MaxEnt Calibration and Evaluation

MaxEnt 3.4.4 was run with logistic output. Candidate settings were calibrated in kuenm version 1.1.10 [7] using the same occurrence records, background sample, and predictors. Terrestrial China was used as the calibration background and geographic projection and management domain because the study objective was national medicinal-resource screening. The background comprised Chinese terrestrial grid cells with complete values for the 9 final predictors: 10,000 cells were sampled randomly from 545,381 valid environmental cells, and MaxEnt additionally incorporated occurrence cells into background feature space. The study did not designate terrestrial China as a biologically delimited accessible area (M) or as the boundary of the species’ ecological niche. Records span multiple regions of China, but dispersal history and colonization barriers are insufficiently known to define a defensible species-specific M—a narrow occurrence buffer could also exclude accessible but unsampled regions. Because background extent can influence response functions, discrimination metrics, and predicted area [33], the broad domain is used only to compare relative suitability for national planning. Its implications and the need for alternative-background sensitivity analyses are stated in the limitations.

Calibration proceeded in two stages. First, 9 RM values (0.1, 0.5, 1, 1.5, 2, 2.5, 3, 3.5, and 4) were crossed with all 31 non-empty combinations of linear (L), quadratic (Q), product (P), threshold (T), and hinge (H) features, yielding 279 initial models. This grid covered a commonly used gradient from weak to strong regularization without excluding a response form a priori. Preliminary test AUC, omission rate, parameter count, and approximate AICc diagnostics were then used to define 48 models for formal reevaluation, including historical reference settings. Formal selection required (1) a significant partial ROC test and (2) a 5% training omission rate ≤ 0.05. AICc and parameter count were compared only among models satisfying both criteria. The selected model, M_0.1_F_lq, used RM = 0.1 and LQ features, had a 5% omission rate of 0.0426, an AICc of 4333.32, and 17 parameters. Diagnostics for the 279 initial, 48 reevaluated, and 3 qualified models are provided in Table S3.

**Figure 2:**
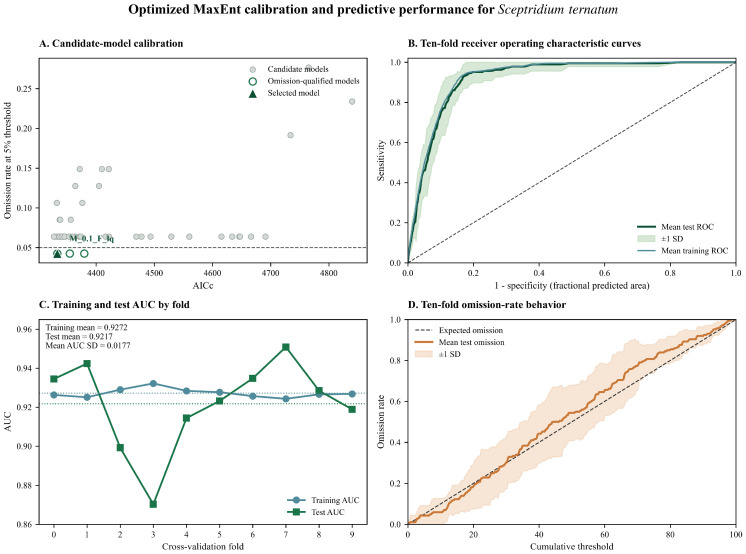
MaxEnt calibration and predictive performance. (**A**) AICc and 5% omission rates of candidate models—the horizontal dashed line marks the 5% omission threshold, and the dark-green triangle identifies M_0.1_F_lq. (**B**) Receiver operating characteristic curves from ten-fold cross-validation; the diagonal dashed line denotes no-skill performance. (**C**) Training and test AUC by fold. (**D**) Ten-fold omission behavior; the diagonal dashed line denotes expected omission.

Ten-fold random cross-validation was performed with the MaxEnt random-seed option and complete fold outputs. Full-sample training AUC described discrimination in the fitted data, whereas mean test AUC described random hold-out performance. To reduce reliance on random partitioning, occurrences were also separated into four spatial blocks using median longitude and latitude, and each block was withheld in turn [13,14]. Test blocks contained 25–69 records, and block-specific training and test sizes and AUC values are reported in Table S3. The continuous Boyce index was calculated from the final full-sample logistic prediction. Background predictions were divided at their deciles into ten approximately equal-frequency bins. For each bin, the ratio of occurrence frequency to background frequency (P/E) was calculated, followed by Spearman correlation between bin midpoints and P/E ratios [11,12]. Bin edges, frequencies, P/E ratios, and the resulting statistic are reported in Table S3. Boyce evaluation supplemented, but did not replace, spatial cross-validation. Although 187 grid-cell occurrences are adequate for model fitting, they remain finite and spatially uneven. Spatial thinning, a 17-parameter final model, and spatial-block validation were used to limit memorization and optimistic evaluation, but they cannot eliminate overfitting or sampling bias. The calibration and validation outputs are summarized in Figure 2.

### 2.4. Suitability Classification and Area Calculation

Suitable and unsuitable cells were separated using the mean maximum training sensitivity plus specificity (MTSPS) threshold from ten-fold cross-validation (0.2233). Suitable cells were further classified as low (0.2233 ≤ *p* < 0.5), moderate (0.5 ≤ *p* < 0.7), or high suitability (*p* ≥ 0.7). The values 0.5 and 0.7 were used only to standardize mapped classes and comparisons among scenarios—they were not interpreted as physiological thresholds. Cell areas in geographic coordinates were corrected by latitude and are reported as 10^4^ km^2^.

### 2.5. Future Climate Scenarios, Range Change, and Centroids

Future climate layers were obtained from downscaled CHELSA CMIP6 products under the bounding SSP1-2.6 and SSP5-8.5 pathways [34,35]. SSP1-2.6 represents a low-forcing pathway with strong mitigation and radiative forcing of approximately 2.6 W m^−2^ by 2100, whereas SSP5-8.5 represents fossil-fuel-intensive development and forcing of approximately 8.5 W m^−2^. These pathways were selected to bracket contrasting climate pressures rather than to identify a single most likely future. We used IPSL-CM6A-LR [36] and MRI-ESM2-0 [37] for 2011–2040, 2041–2070, and 2071–2100 (hereafter the 2040s, 2070s, and 2100s). The two GCMs represent structural differences between climate models. Because they do not constitute a full CMIP6 ensemble, results are reported as their mean and minimum–maximum range.

Only the dynamic climate predictors in the final model were replaced for future projections—topography, soil, NDVI, and UV-B were held constant. This design permits direct climate scenario comparison but does not represent future vegetation, land-use, soil, or radiation dynamics. Current and future binary suitability maps were overlaid to distinguish stable habitat, contraction, and expansion. Geographic centroids were area weighted. Centroid displacement represents change in the spatial center of suitability, not actual population movement.

### 2.6. Post Hoc Habitat and Human-Pressure Overlays

Forest, shrubland, grassland, and wetland were considered land-cover-compatible potential habitat based on the reported shaded-shrub ecology of *S. ternatum* and the need to retain broadly compatible moist vegetation types at the national scale [18]. This operation did not alter MaxEnt training.

Protected-area boundaries were obtained from a 2024 Chinese nature-reserve dataset citing the Chinese Nature Reserve Bio-Specimen Resources Sharing Platform (http://cnpapc.zrbhq.cn/; accessed on 30 June 2026) as its source. Boundaries were projected consistently and converted to the modeling grid using cell centers. Human pressure was represented by the annual global terrestrial Human Footprint dataset of Mu et al. [38], resampled to the modeling grid. Area-weighted HFP was summarized as <10, 10–20, and ≥20. Roads were obtained from 2024 OpenStreetMap data [39]. Motorway, trunk, primary, secondary, tertiary, and corresponding link classes were retained, and Euclidean distance from each grid cell to the nearest major road was calculated. These layers were used only to assess current habitat compatibility, protected-area coverage, relative human pressure, and survey accessibility. They were not treated as MaxEnt predictors or direct causal determinants of occurrence.

### 2.7. Conservation and Survey Priority Rules

Four mutually exclusive management classes were defined: (1) protected highly suitable core habitat: highly suitable, land-cover compatible, and inside a protected area; (2) unprotected low-pressure conservation gap: highly suitable, land-cover compatible, outside protected areas, and HFP < 10; (3) accessible survey priority: moderate or high suitability, land-cover compatible, outside protected areas, HFP < 10, and ≤25 km from a major road; (4) other land-cover-compatible suitable habitat. HFP < 10 and road distance ≤ 25 km were operational decision thresholds, not ecological thresholds. These classes provide regional decision support and do not replace field verification.

## 3. Results

### 3.1. Model Selection and Evaluation

The 279 initial models covered 9 RM values and 31 FC combinations. Forty-eight models proceeded to formal kuenm reevaluation, and three met both the significant partial ROC and 5% omission criteria. M_3_F_qpt had the lowest absolute AICc among all 48 models but an omission rate of 0.0638 and, therefore, failed the prespecified criterion. Among qualified models, M_0.1_F_lq had the lowest AICc and only 17 parameters and was selected as the final model.

Full-sample training AUC was 0.9260. Mean random ten-fold test AUC was 0.9217 with a mean AUC standard deviation of 0.0177. Spatial-block test AUC ranged from 0.8248 to 0.8800 and averaged 0.8575 ± 0.0234. The continuous Boyce index was 0.8211 (*p* = 0.0036). Lower spatial-block than random-fold performance indicates that spatial proximity inflated apparent random-fold accuracy, although discrimination remained good in spatially independent blocks. These evaluation metrics are summarized in Table 2.

### 3.2. Predictor Importance

NDVI, chelsa_gddlgd10, chelsa_prec_10, and chelsa_prec_02 contributed 32.77%, 25.57%, 21.34%, and 8.34%, respectively, for a cumulative contribution of 88.02%. Slope, gypsum content, aluminum saturation, the 5 °C growing-season index, and maximum-season UV-B had smaller contributions.

**Figure 3:**
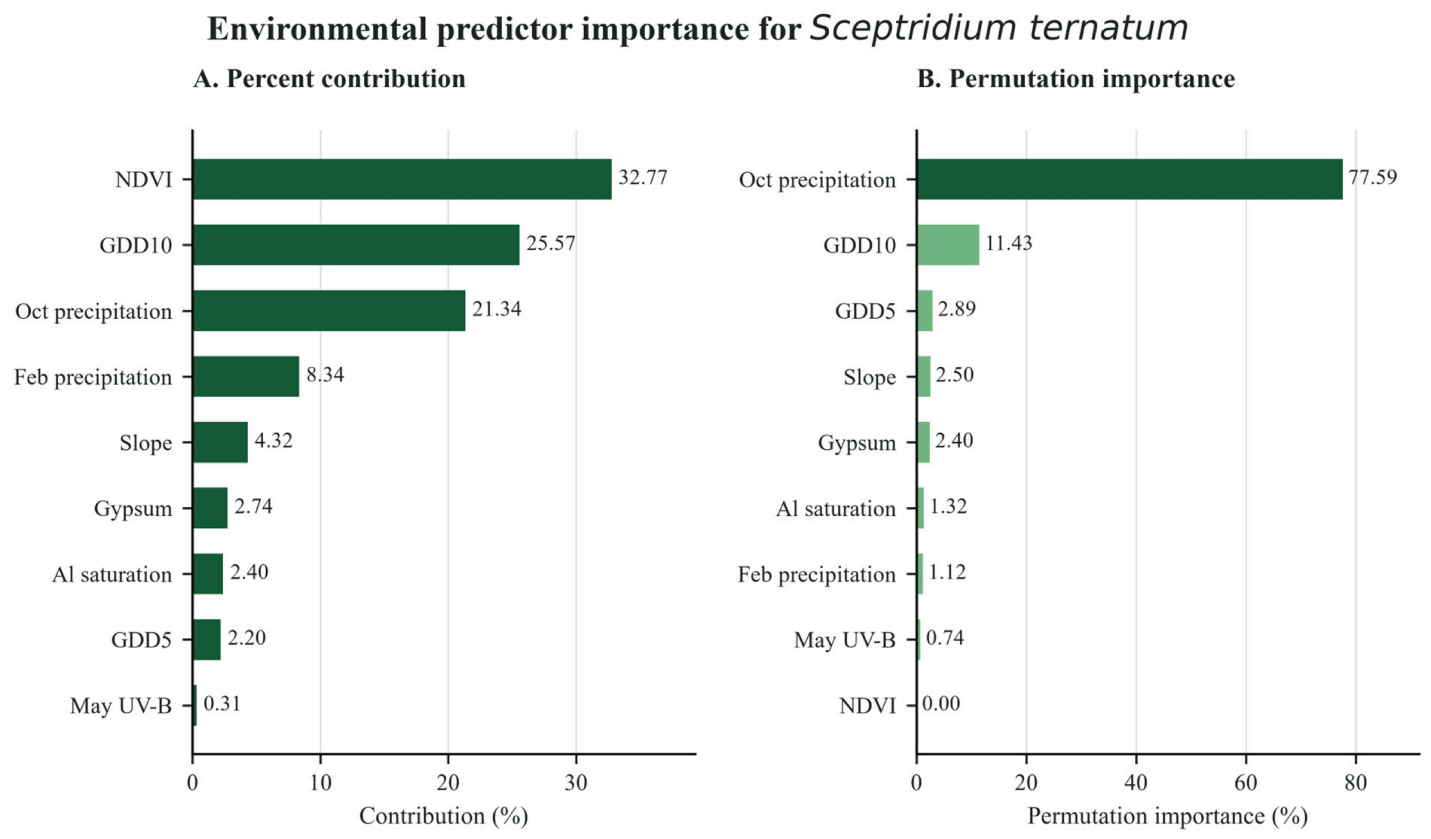
Importance diagnostics for environmental predictors of *Sceptridium ternatum*. (**A**) Percent contribution (dark green). (**B**) Permutation importance; the darkest bar identifies the highest value, and the remaining bars are shown in light green.

Contribution and permutation importance conveyed different information. NDVI had the highest contribution but zero permutation importance, whereas October precipitation had a contribution of 21.34% and permutation importance of 77.59%. NDVI was frequently used during sequential fitting but supplied little unique information after accounting for the other predictors. October precipitation had a much stronger independent influence on predictions. These algorithm-dependent diagnostics were, therefore, interpreted jointly and not as causal effect sizes. The corresponding predictor diagnostics are reported in Table 3 and Figure 3.

### 3.3. Current Potentially Suitable Habitat

**Figure 4:**
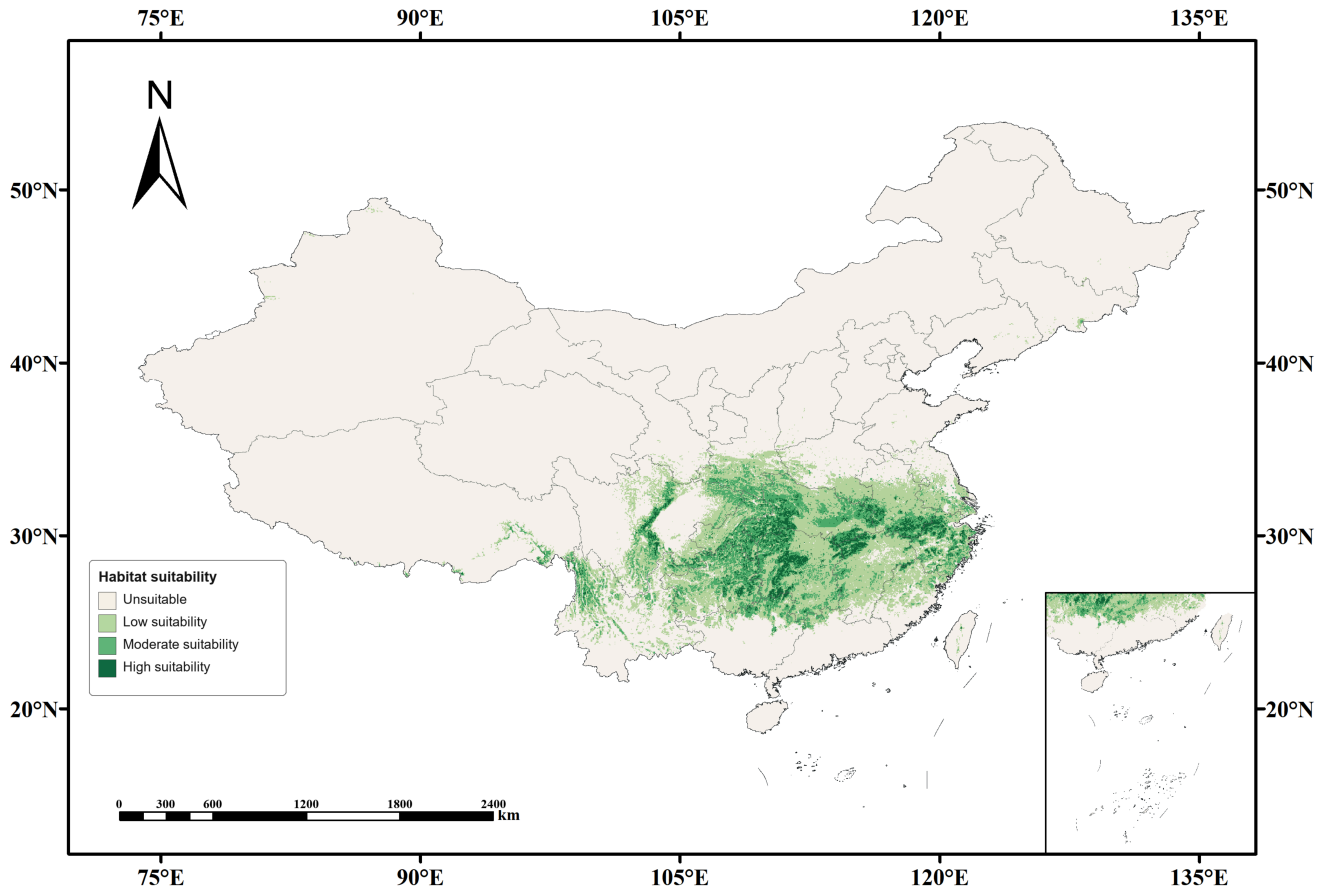
Current potential suitability of *Sceptridium ternatum*. The suitable/unsuitable boundary is the mean MTSPS threshold from ten-fold cross-validation (0.2233). The inset shows the South China Sea islands for cartographic completeness and does not represent a separate study or model area.

Current total suitable habitat covered 157.70 × 10^4^ km^2^, or 16.88% of the valid study area. Low-, moderate-, and high-suitability habitats covered 99.18 × 10^4^, 46.00 × 10^4^, and 12.53 × 10^4^ km^2^, respectively. Moderate and high suitability together covered 58.53 × 10^4^ km^2^. Suitable habitats were concentrated in humid mountains and hills of central, eastern, and southwestern China. Arid northwestern China and the cold, high-elevation Tibetan Plateau were generally unsuitable. Highly suitable patches were relatively concentrated but discontinuous. These class areas and spatial patterns are summarized in Table 4 and Figure 4.

### 3.4. Future Changes in Suitable Habitat

Under SSP1-2.6, mean total suitable habitat in the 2040s, 2070s, and 2100s was 150.07 × 10^4^, 145.46 × 10^4^, and 158.29 × 10^4^ km^2^, representing changes of −4.84%, −7.76%, and 0.37% relative to current conditions. Although the late-century total approached the current area, highly suitable habitat declined to 4.74 × 10^4^, 4.32 × 10^4^, and 4.50 × 10^4^ km^2^ in the three periods.

**Figure 5:**
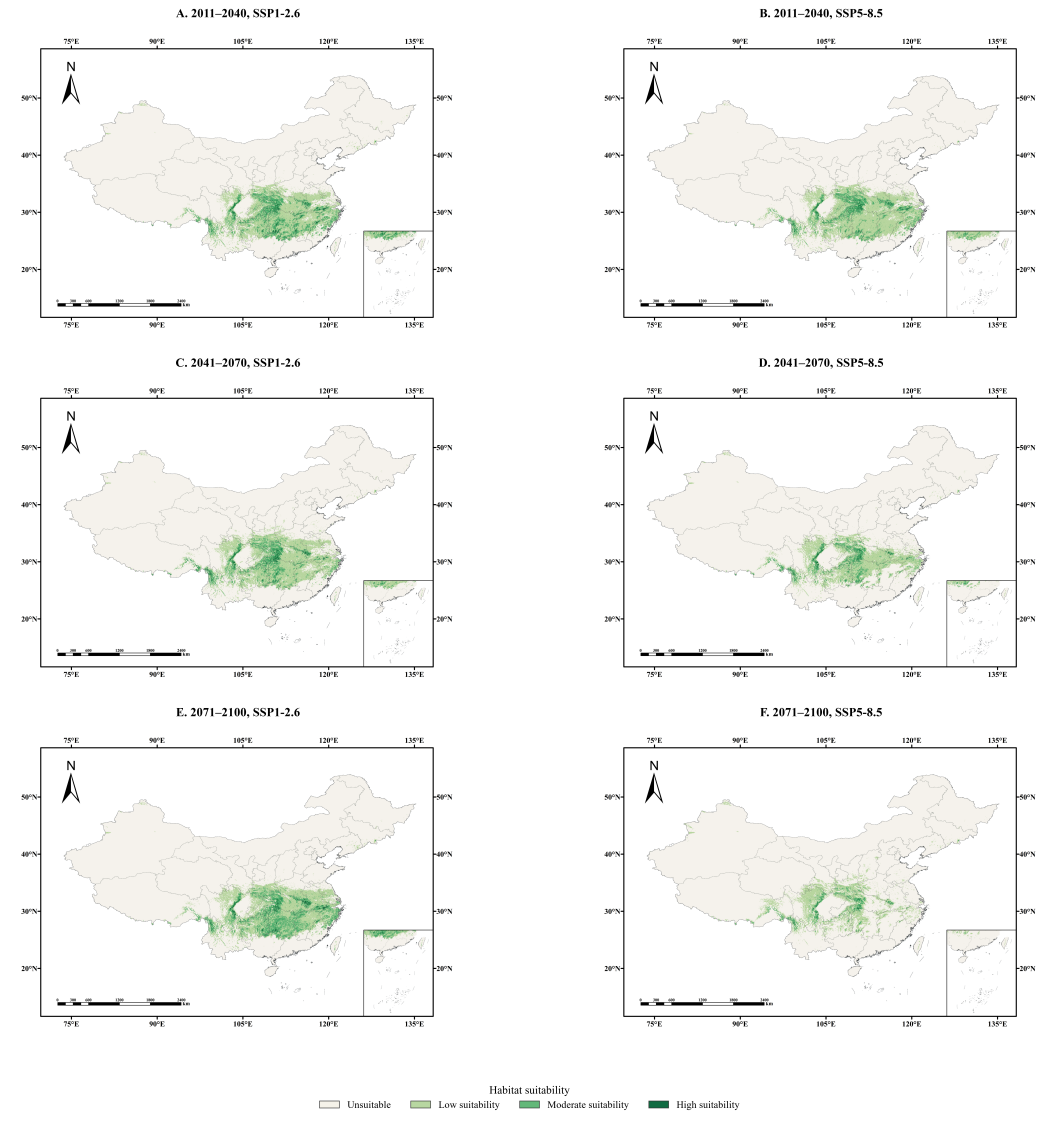
Future potential suitability of *Sceptridium ternatum*. Panels show arithmetic means of IPSL-CM6A-LR and MRI-ESM2-0 under SSP1-2.6 and SSP5-8.5 for the 2040s, 2070s, and 2100s. The insets show the South China Sea islands for cartographic completeness and do not represent separate study or model areas.

Under SSP5-8.5, total suitable habitat decreased progressively to 141.42 × 10^4^, 107.35 × 10^4^, and 86.65 × 10^4^ km^2^. By the 2100s, total suitable habitat was 45.06% below the current area, while highly suitable habitat declined to 1.26 × 10^4^ km^2^, a reduction of 89.96%. The two-GCM range for late-century total suitable habitat under SSP5-8.5 was 66.48 × 10^4^–106.81 × 10^4^ km^2^, indicating substantial model-dependent uncertainty. The scenario-specific areas and maps are summarized in Table 5 and Figure 5.

### 3.5. Habitat Stability, Contraction, Expansion, and Centroid Shifts

**Figure 6:**
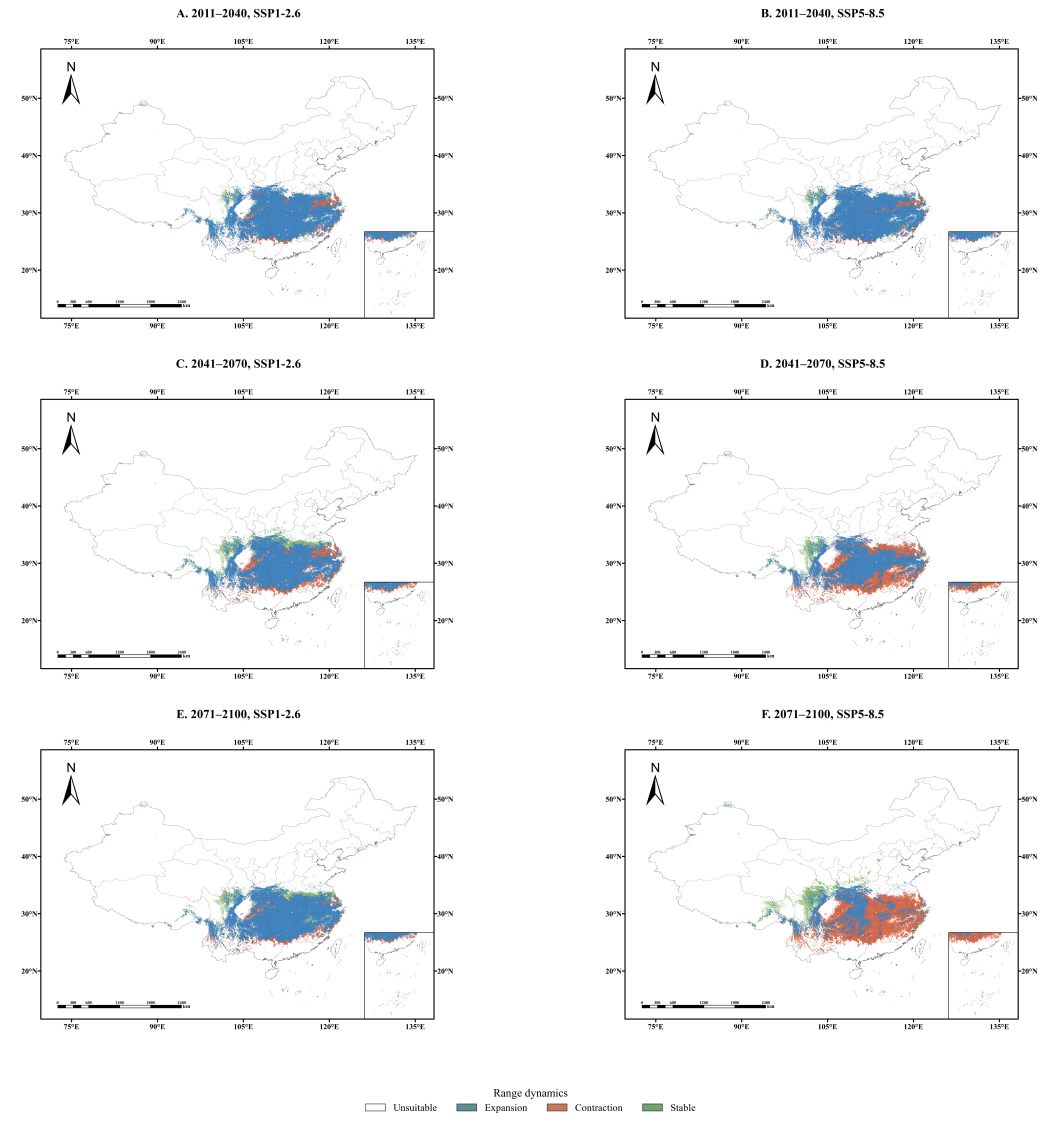
Range dynamics of suitable habitat for *Sceptridium ternatum*, classified as unsuitable area, expansion, contraction, and stable habitat. Each panel compares the current binary map with the mean projection of the two GCMs. The insets show the South China Sea islands for cartographic completeness and do not represent separate study or model areas.

Stable habitat remained extensive under SSP1-2.6. In the 2100s, mean areas of stable habitat, contraction, and expansion were 139.26 × 10^4^, 18.44 × 10^4^, and 19.03 × 10^4^ km^2^, respectively. Contraction increased over time under SSP5-8.5. These range-dynamic patterns are shown in Figure 6. By the 2100s, mean areas of stable habitat, contraction, and expansion were 61.05 × 10^4^, 96.65 × 10^4^, and 25.60 × 10^4^ km^2^.

**Figure 7:**
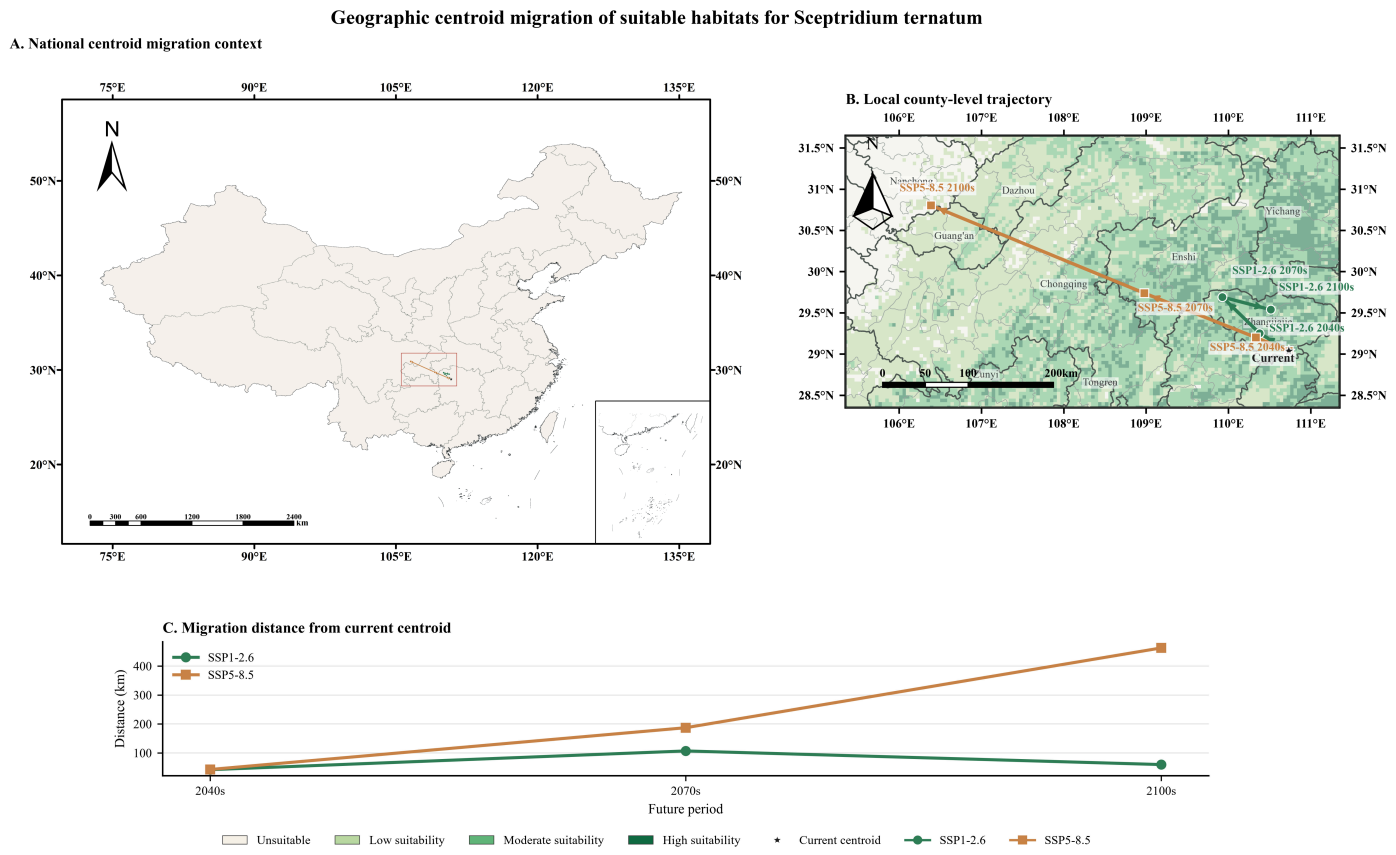
Geographic centroid shifts of suitable habitat. (**A**) National-scale trajectories. (**B**) Enlarged view against prefecture- and county-level administrative boundaries. (**C**) Displacement distance by scenario and period. Lines indicate movement of the spatial center of suitable habitat, not actual population migration. The inset in panel (**A**) shows the South China Sea islands for cartographic completeness and does not represent a separate study or model area.

The current centroid was located near 110.73° E, 29.04° N. Centroid displacement was limited under SSP1-2.6, reaching approximately 59.87 km in the 2100s. Under SSP5-8.5, the mean centroid shifted northwestward by approximately 187 km in the 2070s and 463 km in the 2100s, and the late-century bearing was approximately 296°. These centroid trajectories and distances are shown in Figure 7.

### 3.6. Realistic Habitat and Human-Pressure Constraints

The CLCD overlay identified 100.82 × 10^4^ km^2^ of forest, shrubland, grassland, or wetland within total suitable habitat, representing 63.93% of the total. Land-cover-compatible habitat within highly suitable areas covered 10.40 × 10^4^ km^2^ (82.97%), and forest alone represented 82.38% of highly suitable habitat. Protected areas covered 10.40% of total suitable habitat and 14.77% of highly suitable habitat.

**Figure 8:**
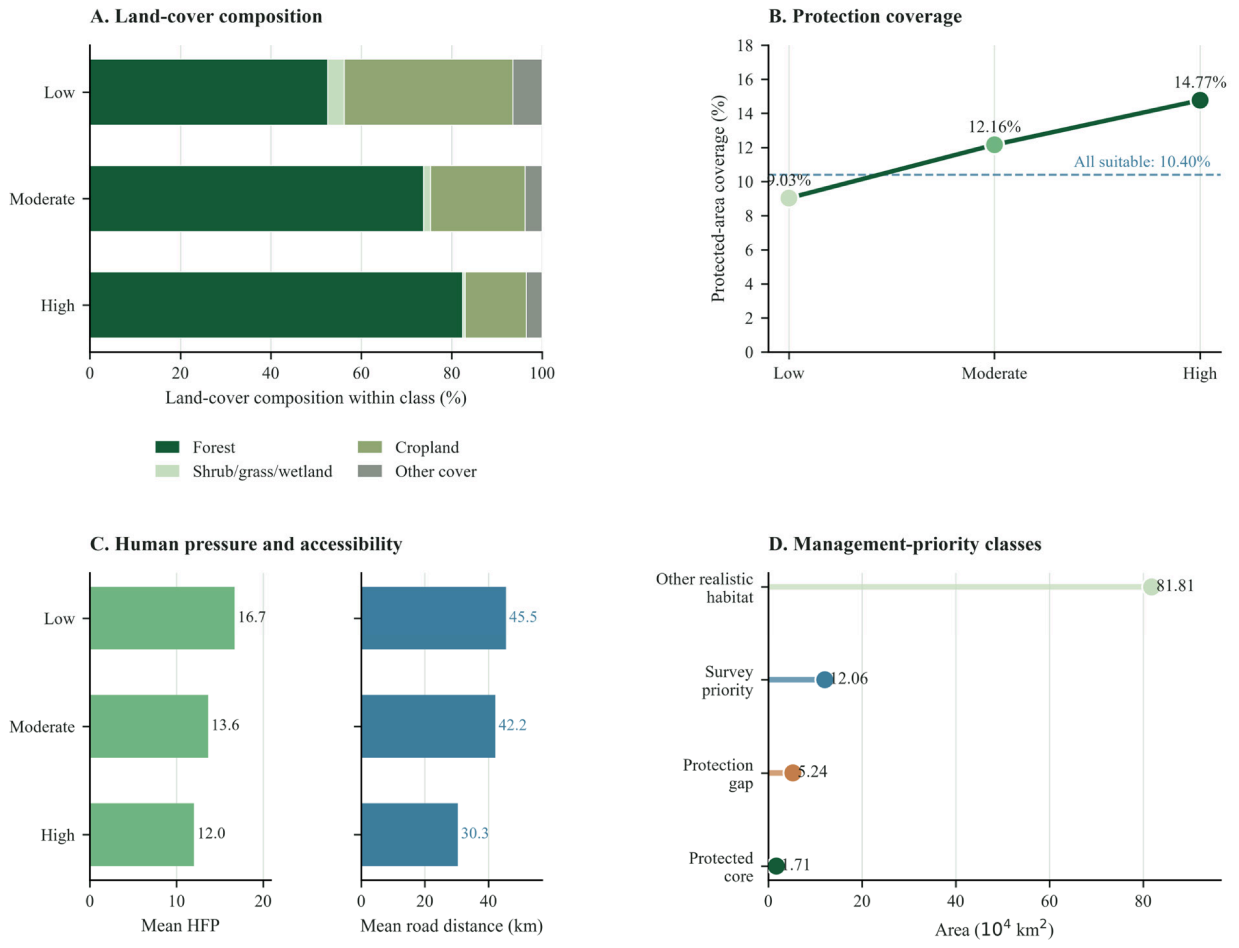
Post hoc habitat and management constraints. (**A**) Land-cover composition by suitability class. (**B**) Protected-area coverage—the dashed line indicates coverage across all suitable habitat. (**C**) Mean human footprint and distance to major roads. (**D**) Areas of mutually exclusive management-priority classes. CLCD, HFP, protected areas, and roads were not used as MaxEnt predictors.

The area-weighted mean HFP in highly suitable habitat was 12.02. HFP classes < 10, 10–20, and ≥20 represented 56.12%, 31.35%, and 12.52% of highly suitable habitat, respectively. Within highly suitable habitat, 82.62%, 1.62%, 1.98%, and 13.77% occurred at distances of ≤10, 10–25, 25–50, and >50 km from major roads. The area-weighted mean road distance was 30.35 km. Road proximity can facilitate field access but may also indicate greater exposure to human activities—accessibility and conservation value were, therefore, interpreted separately. The land-cover, protection, human-pressure, and road-distance summaries are shown in Figure 8.

### 3.7. Conservation and Survey Priorities

**Figure 9:**
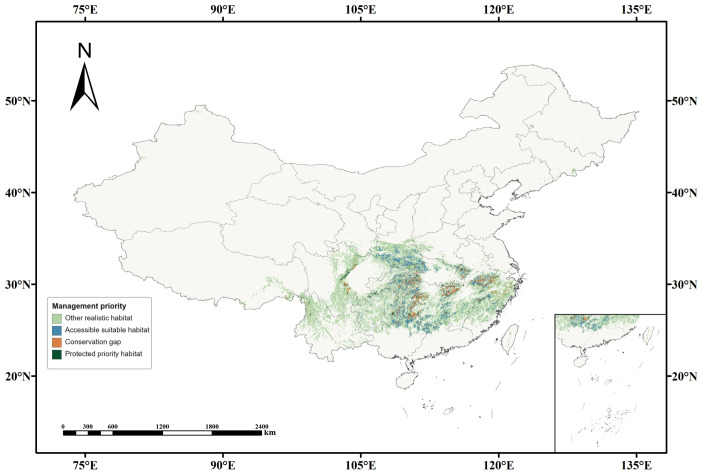
Conservation and survey priority zones for *Sceptridium ternatum*, defined using mutually exclusive rules based on suitability, compatible land cover, protected areas, HFP, and road distance. The inset shows the South China Sea islands for cartographic completeness and does not represent a separate study or model area.

The mutually exclusive classification identified 81.81 × 10^4^ km^2^ of other realistic suitable habitat, 12.06 × 10^4^ km^2^ of accessible survey priority, 5.24 × 10^4^ km^2^ of unprotected low-pressure highly suitable conservation gaps, and 1.71 × 10^4^ km^2^ of protected highly suitable core habitat. Conservation gaps warrant priority verification of wild populations and habitat integrity. Accessible priorities are suited to near-term plot surveys, voucher collection, and germplasm sampling. Protected core habitat is appropriate for long-term monitoring. The complete priority summary is provided in Table 6 and Figure 9.

### 3.8. Inter-Model Uncertainty

**Figure 10:**
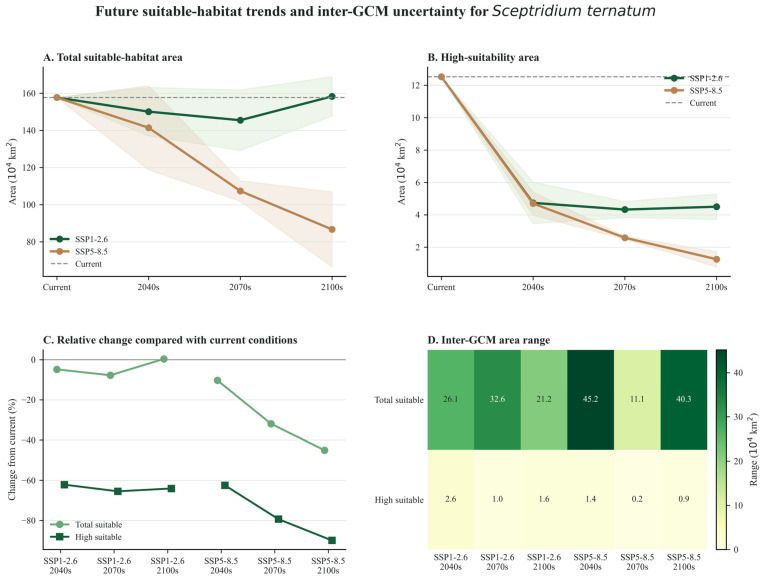
Future suitable-habitat area and inter-model uncertainty. (**A**,**B**) Total and highly suitable habitat—shading indicates the minimum–maximum range of the two GCMs. (**C**) Mean percentage change relative to current conditions. (**D**) Inter-model area range by scenario and period.

The two GCMs agreed on the broad direction of change but differed in magnitude. Under SSP1-2.6, late-century total suitable habitat approached its current area, whereas highly suitable habitat remained below the current area in all periods. Under SSP5-8.5, both total and highly suitable habitats declined over time. Highly suitable habitat was more climate sensitive than total suitable habitat. Inter-model area ranges were largest under late-century high emissions, showing that both emissions pathway and GCM structure affected area estimates. We, therefore, report the two-model mean and minimum–maximum range and do not treat either GCM as a deterministic forecast. The mean trajectories and inter-GCM ranges are shown in Figure 10.

## 4. Discussion

### 4.1. Environmental Basis of Potential Suitability

Seasonal moisture, rather than NDVI, supplied the strongest independent environmental signal. October precipitation had 77.59% permutation importance, consistent with the expectation that late-season moisture may help maintain humid understory conditions for this winter-green fern. The growing-season index at a 10 °C threshold represents the duration of warmer conditions, whereas February precipitation may contribute to early-season soil-water recharge. These are regional associations, not physiological effects, and the response of belowground stages cannot be inferred from the current data.

NDVI had the highest percent contribution but zero permutation importance. Percent contribution is a path-dependent MaxEnt heuristic based on gains added during sequential fitting, whereas permutation importance measures the loss of predictive performance after a fitted variable is randomized. The contrasting values, therefore, indicate that NDVI was used during model fitting but supplied little unique information after the other predictors were considered. Annual maximum NDVI characterizes regional vegetation productivity and cannot be used as a proxy for local shade, canopy openness, forest stratum, or understory moisture. Similarly, the exclusion of elevation at the preliminary contribution screen does not make elevation biologically irrelevant. It indicates that, at this resolution and within this predictor set, elevation added limited algorithmic information and was partly redundant with other regional gradients.

Species biology also identifies processes outside the model. Mycorrhizal associations have been documented in *S. ternatum* roots [20], while subterranean reproductive stages in *Ophioglossaceae* [21] and generation-dependent fungal shifts in *Sceptridium* [22] complicate the interpretation of aboveground occurrences. The model, therefore, estimates regional environmental suitability for recorded sporophytes—it does not represent local fungal partners, demographic state, or establishment probability. Plot-level validation should measure canopy openness, forest or shrub community, soil and litter moisture, substrate, elevation, phenology, population stage, and, where feasible, mycorrhizal context.

### 4.2. Calibration and Predictive Reliability

Default MaxEnt settings can produce overly complex responses and optimistic transfer performance [5,10]. We selected the final model by jointly requiring significant partial ROC, acceptable omission, and favorable AICc and parameter count. M_0.1_F_lq contained only L and Q features and 17 parameters. Although RM = 0.1 is below the MaxEnt default, selection was not based on random AUC alone. The model met the prespecified omission criterion and was evaluated using AICc, model complexity, spatial-block cross-validation, and the Boyce index.

Random-fold test AUC (0.9217) exceeded spatial-block test AUC (0.8575), showing that spatial proximity increased apparent performance under random partitioning. The four spatial blocks also contained unequal numbers of records (25–69), so fold-specific AUC estimates should be interpreted with their sample-size differences in mind. Nevertheless, the range of spatial-block AUC values (0.8248–0.8800) and the positive Boyce index indicated consistent discrimination and suitability ranking across spatially separated subsets. These complementary metrics reduce, but do not eliminate, uncertainty arising from presence-only data and spatial sampling bias.

### 4.3. Climate-Driven Habitat Change

Under SSP1-2.6, total suitable habitat in the 2100s was close to the current area, but highly suitable habitat still declined substantially. Stability in total area, therefore, did not imply stability of core habitat quality, and gains in low-suitability habitat partly offset losses from moderate and high classes. Under SSP5-8.5, late-century total and highly suitable habitats declined by 45.06% and 89.96%, respectively, contraction expanded, and the centroid shifted northwestward.

The GCMs agreed on the overall direction of high-emission change but produced a wide late-century range in total suitable area (66.48 × 10^4^–106.81 × 10^4^ km^2^). This spread reflects uncertainty in regional temperature and precipitation simulated by different climate models. Areas remaining suitable across scenarios and periods may represent potential climatic refugia, but their practical value depends on land cover, human pressure, existing populations, and habitat condition.

### 4.4. Realistic Habitat, Conservation Gaps, and Survey Priorities

Compatible land-cover classes represented 63.93% of total suitable habitat, indicating that more than one-third of environmentally suitable cells did not match the broad habitat types considered usable by this understory fern. Forest dominated highly suitable habitat, consistent with published records that associate the species with shaded habitats [18]. This agreement supports the ecological plausibility of the mapped core area but does not validate local occupancy.

Protected areas covered only 14.77% of highly suitable habitat. Moreover, 43.88% of highly suitable habitat had HFP ≥ 10 and 12.52% had HFP ≥ 20, showing that environmental suitability often coincided with non-negligible human pressure. The unprotected, low-pressure, highly suitable class should, therefore, be prioritized for population verification and local conservation assessment. Protected core habitat provides candidates for long-term monitoring and germplasm conservation. Road-proximate areas may be efficient for initial surveys, but accessibility should not be conflated with low disturbance or high conservation value. Our mutually exclusive classes separate survey efficiency from conservation urgency.

### 4.5. Practical Recommendations and Methodological Outlook

The transferable value of this workflow lies not in a new MaxEnt algorithm, but in separating three questions that are often conflated in distribution studies: where regional environmental conditions are suitable, whether the mapped area contains compatible contemporary habitat, and whether it is appropriate for conservation action or efficient field survey. This separation is relevant to other data-limited understory medicinal plants for which coarse climatic suitability, realized habitat, and management feasibility do not necessarily coincide.

The four priority classes support different actions. Protected highly suitable sites are candidates for repeated, seasonally standardized population monitoring and habitat-condition assessment. Unprotected, low-pressure, highly suitable gaps should first receive voucher-supported population verification, followed by local protection or habitat-management evaluation where persistent populations are confirmed. Road-accessible priorities are appropriate for efficient reconnaissance and germplasm collection, but road proximity is not evidence of conservation value. At every confirmed site, field teams should record canopy openness, vegetation stratum and associated species, soil or litter moisture, substrate, elevation, phenological stage, population size or stage structure, disturbance, and collection pressure.

Methodological improvement should focus on the assumptions most relevant to transfer. Future studies should compare alternative biologically informed calibration backgrounds, test elevation- and canopy-related variables when reliable layers or field measurements are available, evaluate multiple SDM algorithms and a larger GCM ensemble, and incorporate dynamic land use where possible. Repeated observations and targeted surveys in predicted gaps would also permit occupancy or abundance modeling and provide a stronger test of whether the present suitability classes predict population persistence.

### 4.6. Limitations

This study uses 1 presence-background algorithm and 187 retained grid-cell occurrences, including some records georeferenced from locality descriptions. The transparent 104-to-11-to-9 screening log improves reproducibility, but the initial > 1.3% contribution rule was data driven and may omit ecologically meaningful variables. The national calibration background was selected for national resource planning, not as a biologically delimited M, and alternative background definitions may change response functions and discrimination metrics. Parameter calibration, spatial thinning, partial ROC, omission rate, AICc, spatial-block validation, and the Boyce index reduce, but do not remove, overfitting and spatial sampling bias.

The 2.5 arc-min predictors do not resolve local shade, canopy structure, associated plant communities, soil moisture, fungal partners, or demographic state. Future projections used only two GCMs and two SSPs and held soil, topography, NDVI, and UV-B constant. They, therefore, represent climate-conditioned screening rather than forecasts of future vegetation, land use, dispersal, harvesting, or population dynamics.

The land-cover, protected-area, human footprint, and road overlays translate suitability into operational priorities but do not establish causal effects on occurrence. The mapped classes should guide where to verify populations and collect standardized field evidence—they should not replace site-level conservation assessment.

## 5. Conclusions

This study achieved its objective of identifying national-scale current and future suitability for *Sceptridium ternatum* and separating conservation needs from survey accessibility. The results support the working expectations that seasonal moisture provides the strongest independent regional signal, that highly suitable habitat is more climate sensitive than total suitable habitat under high emissions, and that existing protected areas leave substantial highly suitable habitat outside formal coverage. The analysis, therefore, advances species-specific planning beyond a suitability map by combining calibrated model evaluation, inter-GCM uncertainty, land-cover compatibility, human pressure, protected areas, and access constraints. Its broader relevance is the explicit separation of regional suitability, contemporary habitat compatibility, and management feasibility for data-limited understory medicinal plants.

Protected core sites should be monitored repeatedly, unprotected low-pressure gaps should receive priority population verification and local conservation assessment, and accessible areas should be used for efficient voucher and germplasm surveys. These recommendations remain conditional on field confirmation because the 187 records are spatially uneven, the calibration background is broad, and 5 km predictors cannot represent shade, soil moisture, demographic stages, or mycorrhizal context. Future work should combine standardized field measurements with alternative calibration backgrounds, additional climate models and SDM algorithms, and dynamic land-use information.

## Figures and Tables

**Table 1:** Candidate predictor groups and variables retained in the final MaxEnt model.

Predictor Group	Candidate Variables (*n*)	Retained (*n*)	Predictors in the Final Model	Main Source
CHELSA bioclimatic variables	19	0	None	CHELSA v2.1
CHELSA monthly climate variables	48	2	chelsa_prec_02; chelsa_prec_10	CHELSA v2.1
Growing-degree-day and frost indices	7	2	chelsa_gddlgd5; chelsa_gddlgd10	CHELSA-BIOCLIM+ v2.1
Topographic variables	3	1	slope	WorldClim v2.1 DEM; derived in ArcGIS
Soil and substrate attributes	20	2	gypsum; alum_sat	HWSD v1.2
Vegetation index	1	1	ndvi	China 30 m annual maximum NDVI dataset
UV-B radiation variables	6	1	uvb5	glUV
Total	104	9	Nine predictors used for modeling	Full inventory in Table S2

**Table 2:** Evaluation metrics for the optimized MaxEnt model.

Metric	Value	Interpretation
Partial ROC *p*-value	<0.001	Candidate-model significance
5% omission rate	0.0426	Parameter-screening criterion
AICc	4333.32	Lowest among qualified models
Number of parameters	17	RM = 0.1; FC = LQ
Full-sample training AUC	0.9260	Full-sample fit
Mean random ten-fold test AUC	0.9217; mean AUC SD = 0.0177	Random hold-out validation
Spatial-block test AUC	0.8575 ± 0.0234	Four-fold spatial validation
Continuous Boyce index	0.8211	*p* = 0.0036

**Table 3:** Importance of predictors in the final model.

Predictor	Description	Contribution (%)	Permutation Importance (%)
ndvi	Normalized difference vegetation index	32.77	0.00
chelsa_gddlgd10	Growing-season index at a 10 °C threshold	25.57	11.43
chelsa_prec_10	October precipitation	21.34	77.59
chelsa_prec_02	February precipitation	8.34	1.12
slope	Slope	4.32	2.50
gypsum	Soil gypsum content	2.74	2.40
alum_sat	Soil aluminum saturation	2.40	1.32
chelsa_gddlgd5	Growing-season index at a 5 °C threshold	2.20	2.89
uvb5	Maximum-season UV-B	0.31	0.74

**Table 4:** Current habitat suitability classes for *Sceptridium ternatum*.

Class	Suitability Range	Area (10^4^ km^2^)	Valid Study Area (%)
Unsuitable	<0.2233	776.44	83.12
Low suitability	0.2233–0.5	99.18	10.62
Moderate suitability	0.5–0.7	46.00	4.92
High suitability	≥0.7	12.53	1.34
Total suitable	≥0.2233	157.70	16.88

**Table 5:** Future suitable-habitat area averaged across two global climate models.

Scenario	Period	Total Suitable (10^4^ km^2^)	Change in Total (%)	Moderate + High (10^4^ km^2^)	High Suitability (10^4^ km^2^)
SSP1-2.6	2011–2040	150.07	−4.84	44.76	4.74
SSP1-2.6	2041–2070	145.46	−7.76	39.88	4.32
SSP1-2.6	2071–2100	158.29	0.37	51.22	4.50
SSP5-8.5	2011–2040	141.42	−10.32	43.85	4.70
SSP5-8.5	2041–2070	107.35	−31.93	26.88	2.59
SSP5-8.5	2071–2100	86.65	−45.06	16.71	1.26

**Table 6:** Main post hoc habitat-constraint and management-priority results.

Metric	Value	Unit or Proportion
Land-cover-compatible habitat within total suitable habitat	100.82	10^4^ km^2^ (63.93%)
Land-cover-compatible habitat within highly suitable habitat	10.40	10^4^ km^2^ (82.97%)
Protected-area coverage of total suitable habitat	10.40	%
Protected-area coverage of highly suitable habitat	14.77	%
Highly suitable habitat with HFP < 10	56.12	%
Highly suitable habitat with HFP ≥ 20	12.52	%
Highly suitable habitat ≤ 10 km from a major road	82.62	%
Unprotected low-pressure highly suitable conservation gap	5.24	10^4^ km^2^
Accessible survey priority	12.06	10^4^ km^2^
Protected highly suitable core habitat	1.71	10^4^ km^2^

## Data Availability

The retained occurrence records, predictor inventory, initial and reevaluated model diagnostics, qualified-model table, principal area summaries, and data source inventory are provided in the Supplementary Materials. The GBIF records are available under download DOI 10.15468/dl.7fwvw5. Records from CVH, PlantPlus, the National Plant Specimen Resource Center, ACM, the Fourth National Survey of Chinese Materia Medica Resources, and field investigations are identified by source category in Table S1. The original public environmental datasets can be obtained from the providers listed in Table S6. Derived statistics used in the manuscript are included in the Supplementary Materials. OpenStreetMap-derived data are used under the Open Database License.

## Supplementary Materials

The following supporting information can be downloaded at: https://www.mdpi.com/article/10.3390/biology15151268/s1. Table S1: Retained occurrence records and source categories. Table S2: Inventory, sources, preliminary contributions, screening decisions, final status, correlation matrix, and variance inflation factors for the retained environmental predictors. Table S3: Initial-model diagnostics, formal reevaluation results, qualified models, spatial-block validation results, and continuous Boyce-index bin calculations. Table S4: Current suitable-habitat area. Table S5: Multi-GCM future suitable-habitat area. Table S6: Data source and licensing inventory. The GBIF records correspond to download DOI 10.15468/dl.7fwvw5.
